# Comparative Transcriptome Analyses Reveal Potential Mechanisms of Enhanced Drought Tolerance in Transgenic *Salvia Miltiorrhiza* Plants Expressing AtDREB1A from Arabidopsis

**DOI:** 10.3390/ijms19030827

**Published:** 2018-03-12

**Authors:** Tao Wei, Kejun Deng, Hongbin Wang, Lipeng Zhang, Chunguo Wang, Wenqin Song, Yong Zhang, Chengbin Chen

**Affiliations:** 1 National Engineering Research Center of Pesticide (Tianjin), Nankai University, Tianjin 300071, China; 8190537@nankai.edu.cn; 2 College of Life Sciences, Nankai University, Tianjin 300071, China; 2120150901@mail.nankai.edu.cn (H.W.); 1120140344@mail.nankai.edu.cn (L.Z.); wangcg@nankai.edu.cn (C.W.); songwq@nankai.edu.cn (W.S.); 3 School of Life Sciences and Technology, University of Electronic Science and Technology of China, Chengdu 610054, China; dengkj@uestc.edu.cn; 4 Center for Informational Biology, University of Electronic Science and Technology of China, Chengdu 610054, China

**Keywords:** transcriptome, drought, *AtDREB1A*, *Salvia miltiorrhiza*, phytohormones, transcription factors, photosynthesis

## Abstract

In our previous study, drought-resistant transgenic plants of *Salvia miltiorrhiza* were produced via overexpression of the transcription factor AtDREB1A. To unravel the molecular mechanisms underpinning elevated drought tolerance in transgenic plants, in the present study we compared the global transcriptional profiles of wild-type (WT) and *AtDREB1A*-expressing transgenic plants using RNA-sequencing (RNA-seq). Using cluster analysis, we identified 3904 differentially expressed genes (DEGs). Compared with WT plants, 423 unigenes were up-regulated in pRD29A::AtDREB1A-31 before drought treatment, while 936 were down-regulated and 1580 and 1313 unigenes were up- and down-regulated after six days of drought. COG analysis revealed that the ‘signal transduction mechanisms’ category was highly enriched among these DEGs both before and after drought stress. Based on the Kyoto Encyclopedia of Genes and Genomes (KEGG) annotation, DEGs associated with “ribosome”, “plant hormone signal transduction”, photosynthesis”, “plant-pathogen interaction”, “glycolysis/gluconeogenesis” and “carbon fixation” are hypothesized to perform major functions in drought resistance in *AtDREB1A*-expressing transgenic plants. Furthermore, the number of DEGs associated with different transcription factors increased significantly after drought stress, especially the AP2/ERF, bZIP and MYB protein families. Taken together, this study substantially expands the transcriptomic information for *S. miltiorrhiza* and provides valuable clues for elucidating the mechanism of AtDREB1A-mediated drought tolerance in transgenic plants.

## 1. Introduction

RNA-sequencing (RNA-seq) is a powerful analytical tool for transcriptomic studies. It is a well-established method that continues to develop rapidly, driving down the cost of data acquisition and helping to unravel complex gene expression and regulation phenomena [1,2,3]. This technology has been applied recently to diverse plant species to examine the genetic responses to abiotic and biotic stresses such as drought, high and low temperatures, salinity, sulfate starvation, nitrogen deficiency and pathogen infection, as well as changes in gene expression occurring during development [4,5,6,7,8,9,10,11,12,13]. Garg et al. [8] performed RNA-seq analyses in salt-tolerant and drought-related chickpea genotypes under normal and stressed conditions during vegetative and reproductive growth. Comparative transcriptomics revealed differences in gene expression between the two genotypes during different developmental stages. In all, 5545 and 4954 genes were regulated only in salt- and drought-tolerant genotypes, respectively [8]. In maize, RNA-seq was performed to measure changes in the global transcriptome before and after freezing treatment. This study identified 948 genes that showed differential expression between highly sensitive and highly tolerant lines under freezing conditions. Gene ontology (GO) classifications found that these differentially expressed genes (DEGs) were significantly enriched for binding functions (metal ion binding, DNA binding and ATP binding), peptidase activity and protein kinase activity [6]. In the RNA-seq database of developing canola embryos, Deng et al. [7] identified 55 DEGs encoding 28 enzymatic functions related to carbon flux through fatty acids for the storage of triacylglycerols, 122 DEGs encoding transcription factors and 41 DEGs associated with signal transduction, transport and metabolism of different phytohormones [7].

Recently, transcriptome profiling has been used as a comprehensive non-targeted approach to examine secondary effects on gene expression resulting from the introduction of transgenes [14,15,16,17,18,19,20]. Transcriptomic studies indicated that overexpression of OsiSAP1 in transgenic rice affected the expression of endogenous genes encoding membrane transporters, transcription factors, signaling components, as well as genes involved in growth, development and metabolism. In all, 150 genes were up-regulated in transgenic plants, of which 43 have been linked to stress responses [21]. Transgenic alfalfa plants overexpressing MsmiR156, a precursor of microRNA156, exhibited a reduction in internodal length, as well as increased shoot branching and trichrome density, enhanced biomass yield and delayed flowering. A total of 160,472 transcripts were obtained after RNA sequencing on the Illumina HiSeq2500 and 4985 significantly differentially expressed genes were identified in transgenic miR156OE plants. The enriched GO terms “electron transporter”, “chitin binding”, ‘sucrose-phosphate synthase activity”, “sucrose transport”, ‘sexual reproduction”, “starch synthase activity”, “lignin catabolism”, and “flavonoid biosynthesis” correlate well with the phenotypes of the miR156OE alfalfa plants [3]. In birch, overexpression of the endogenous *Betula platyphylla* APETALA1 (*BpAP1*) gene caused early flowering and 166 putative target genes of *BpAP1* were predicted after combining the RNA-seq database with birch genome information [22]. 

*Salvia miltiorrhiza* Bunge (Lamiaceae)—also known as Chinese sage—is an important herb in Traditional Chinese Medicine (TCM); its rhizome/roots have been widely used for the treatment of various cardiovascular and cerebrovascular diseases [23]. Recently, the transcriptome research on *S. miltiorrhiza* has been mainly focused on the analysis and regulation of the metabolic pathways of tanshinone and salvianolic acid. Arabidopsis transcription factor DREB1A (AtDREB1A) has been proved to be endowed with abiotic stress tolerance in many plant species, such as rice [24], tobacco [25] and potato [26] and many downstream genes have been identified in Arabidopsis, including *rd29A*, *kin1*, *cor15a* and *cor47* [27]. In our previous study, we engineered drought tolerance in *S. miltiorrhiza* by overexpressing *AtDREB1A* [23]. Compared with wild-type (WT), a higher chlorophyll (Chl) content and relative water content (RWC) and an elevated photosynthetic rate were detected in transgenic plants after drought treatment. The MDA content was generally lower in *AtDREB1A* transgenic plants but SOD, CAT and POD activities were higher following drought stress. In addition, we identified some genes (e.g., *PBS1*, *KIN10*, *bHLH122*, *ERF1B* and *LEA*) regulated by AtDREB1A through the analysis of the transcriptome of transgenic plants after drought stress [23]. However, the molecular basis of this elevated drought tolerance and the associated metabolic regulatory pathways remain poorly understood.

In order to further investigate the AtDREB1A-mediated drought tolerance in *S. miltiorrhiza,* we evaluated global changes in gene expression using RNA-sequencing. RNA-seq analysis was performed in a pRD29A::AtDREB1A transgenic line and compared to WT. The results suggest that, even without a stress stimulus, expression of genes associated with plant growth and development was significantly altered in the transgenic line. After exposure to drought stress, the differentially expressed genes (DEGs) related to transcription factors and plant hormone signaling increased significantly. The main pathways regulated by AtDREB1A, based on Kyoto Encyclopedia of Genes and Genomes (KEGG) enrichment analysis, include “ribosome”, “plant hormone signal transduction”, “photosynthesis”, “plant–pathogen interactions”, “glycolysis/gluconeogenesis”, “carbon fixation”, and other metabolic pathways.

## 2. Results

### 2.1. Illumina Sequencing and De Novo Assembly

In an attempt to elucidate the molecular mechanisms underpinning the enhanced drought tolerance of the AtDREB1A overexpression lines, we compared global gene expression profiles of WT and pRD29A::AtDREB1A-31 lines following drought treatments. RNA samples from three separate 25-day-old plants before drought treatment (BD) and 31-day-old plants after 6 days of drought (AD) were combined and used to generate cDNA libraries, yielding 27,026,927, 23,323,827, 23,222,857 and 17,566,704 clean reads for WT (BD), pRD29A::AtDREB1A-31 (BD), WT (AD) and pRD29A::AtDREB1A-31 (AD) cDNA libraries, respectively. The guanine+cytosine (G + C) content in these libraries was 49.35%, 49.90%, 48.74% and 49.75%, respectively and all Q30 percentages were >87% (Table S1). Because no reference genome is available for *S. miltiorrhiza*, a de novo transcriptome assembly was constructed by combining clean reads from all four cDNA libraries. High-throughput RNA sequencing yielded 12,030,368 contigs, 173,129 transcripts and 78,915 unigenes with N50 lengths of 42, 2048 and 1301 nt, respectively (Table S2).

### 2.2. Gene Annotation and Functional Classification of the DEGs

Approximately 48% (37,979) of the assembled unigenes could be annotated by BLAST searches, using a threshold of 1 × 10^−5^, against the COG, KOG, GO, KEGG, Swiss-Prot, Pfam and NCBI nr public databases. Among the annotated unigenes, 13,904 (17.62%), 22,861 (28.97%), 27,694 (35.09%), 10,017 (12.69%), 26,052 (33.01%), 26,117 (33.10%) and 37,091 (47.0%) were annotated using the COG, KOG, GO, KEGG, Swiss-Prot, Pfam and NCBI nr databases, respectively (Table S3).

**Figure 1:**
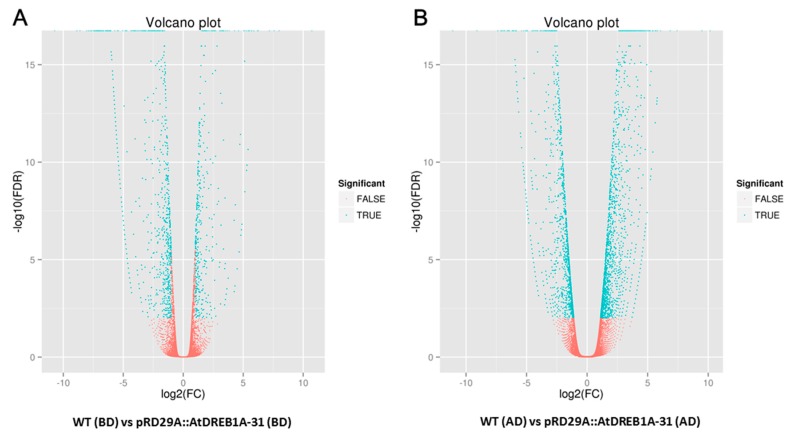
Volcano plots of differentially expressed genes (DEGs) in the comparisons of *AtDREB1A*-overexpressing and wild-type (WT) plants of *Salvia miltiorrhiza* before and after drought treatment. (**A**) WT (BD) versus pRD29A::AtDREB1A-31 (BD); (**B**) WT (AD) versus pRD29A::AtDREB1A-31 (AD). WT, wild type plants of *Salvia miltiorrhiza*; pRD29A::AtDREB1A-31, pRD29A::AtDREB1A transgenic line 31. BD, before drought; AD, after six days of drought treatment.

**Figure 2:**
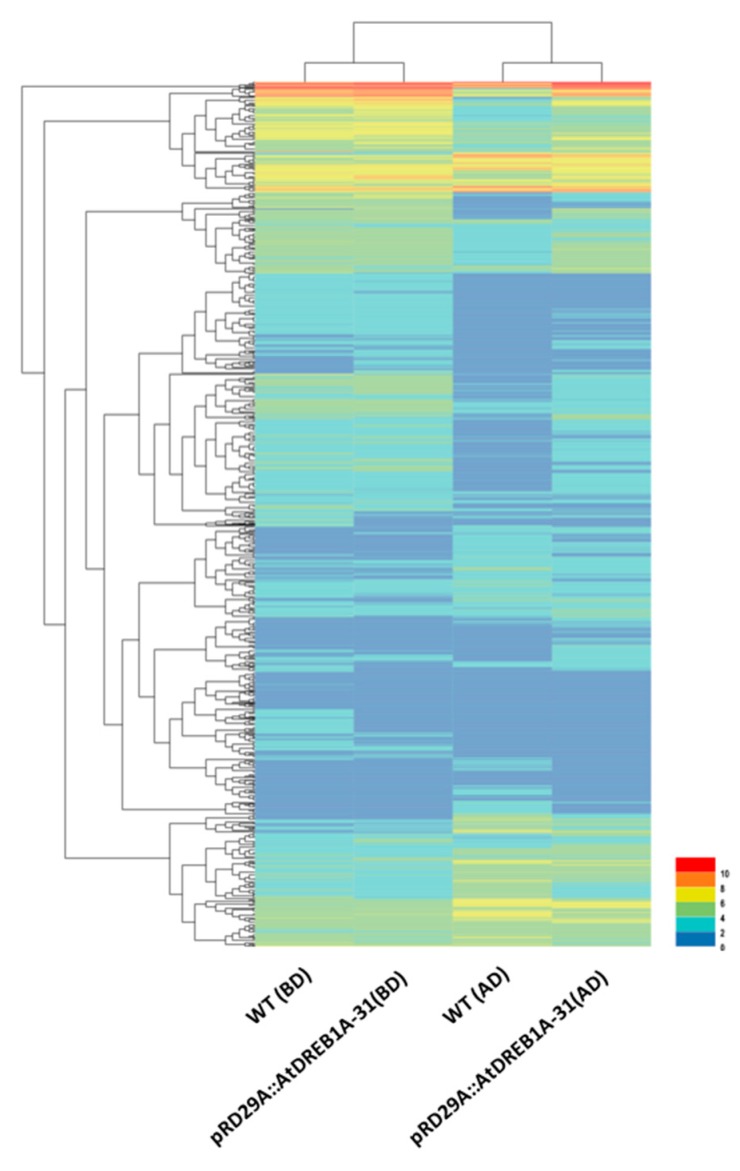
Cluster analysis of DEGs in WT and *AtDREB1A*-expressing transgenic *Salvia miltiorrhiza* plants based on expression profiles measured by RNA-seq. WT, wild type; pRD29A::AtDREB1A-31, pRD29A::AtDREB1A transgenic line 31. BD, before drought; AD, after six days of drought. The color scale in the heat map corresponds to log2 (FPKM) values of genes in various samples.

**Figure 3:**
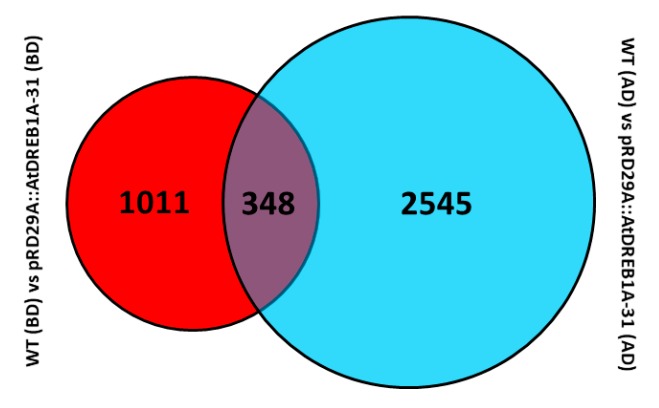
Venn diagram analysis of DEGs between two comparisons (WT (BD) versus pRD29A::AtDREB1A-31 (BD) and WT (AD) versus pRD29A::AtDREB1A-31 (AD)). WT, wild type; pRD29A::AtDREB1A-31, pRD29A::AtDREB1A transgenic line 31. BD, before drought; AD, after six days of drought.

We analyzed unigene expression in WT and *AtDREB1A*-expressing plants using Bowtie and RSEM software and normalized the values using Fragments Per Kilobase Million (FPKM). Differential expression was assigned when (i) FDR values were <0.01; and (ii) the fold-change (FC) was ≥2. DEGs expressed at higher levels in transgenic plants were defined as up-regulated and DEGs expressed at lower levels in transgenic plants were assigned as down-regulated. To determine the number of differentially regulated genes, log2FC values were plotted against negative log10(FDR) to generate a volcano plot (Figure 1). The higher the value of the negative log10(FDR), the more significant the regulation and an log2FC of zero lies in the middle of the volcano, while negative log2FC values on the left indicate a down-regulation and positive log2FC values on the right indicate an up-regulation. A total of 3904 DEGs were identified through volcano plot and cluster analysis (Figure 2), 348 of which were the same in the following comparisons; (1) WT (BD) versus pRD29A::AtDREB1A-31 (BD); and (2) WT (AD) versus pRD29A::AtDREB1A-31 (AD) (Figure 3). Compared with WT, 423 unigenes were up-regulated and 936 were down-regulated in pRD29A::AtDREB1A-31 before drought, while 1580 unigenes were up-regulated and 1313 were down-regulated in pRD29A::AtDREB1A-31 after six days of drought treatment (Table S4).

**Figure 4:**
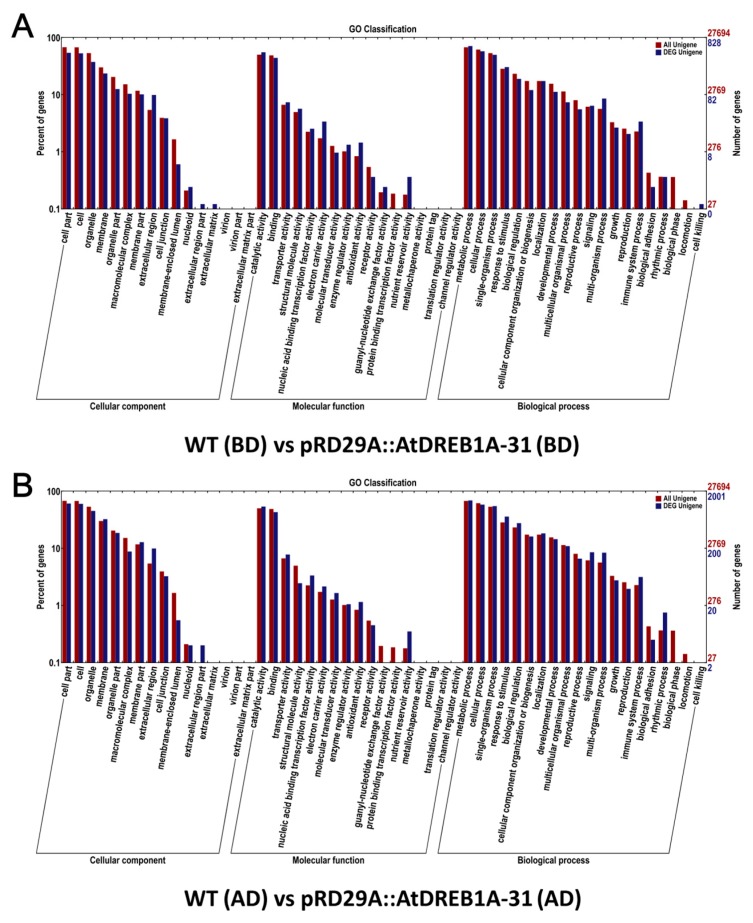
Functional Gene Ontology (GO) term classifications of DEGs in the comparisons between WT and *AtDREB1A* transgenic plants before and after drought treatment. (**A**) WT (BD) versus pRD29A::AtDREB1A-31 (BD); (**B**) WT (AD) versus pRD29A::AtDREB1A-31 (AD). WT, wild type; pRD29A::AtDREB1A-31, pRD29A::AtDREB1A transgenic line 31. BD, before drought; AD, after six days of drought.

Global functional analysis of DEGs was carried out using GO annotation with Blast2GO to derive “cellular component”, “molecular function”, and “biological process” categories (Figure 4). In both comparisons, the most enriched terms for “cellular component” were “cell part”, ‘cell”, and “organelle”, while the dominant categories for ‘molecular function” were “catalytic activity” and “binding”. The “metabolic process” term was the most frequent in the “biological process” category, followed by “cellular process” and “single-organism process” (Figure 4).

### 2.3. COG Enrichment and KEGG Pathway Analysis of DEGs

**Figure 5:**
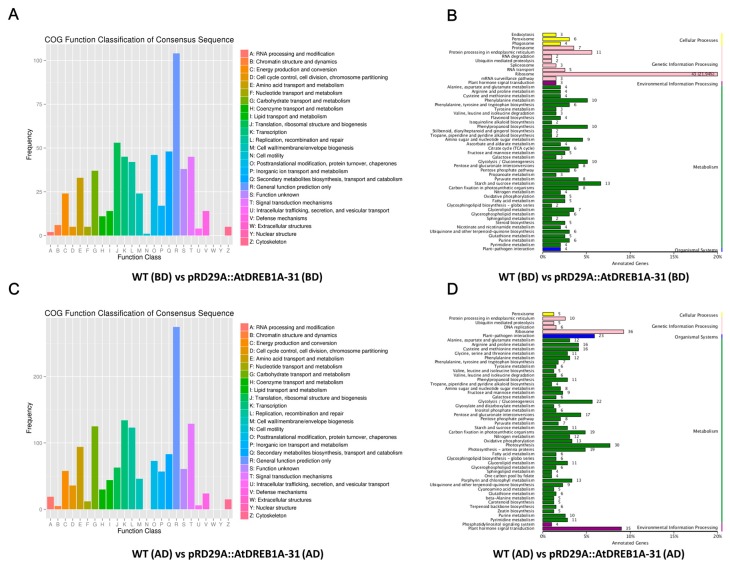
COG and Kyoto Encyclopedia of Genes and Genomes (KEGG) classification of DEGs in the comparisons of WT and *AtDREB1A* transgenic plants before and after drought treatment. (**A**,**B**) WT (BD) versus pRD29A::AtDREB1A-31 (BD); (**C**,**D**) WT (AD) versus pRD29A::AtDREB1A-31 (AD). WT, wild type; pRD29A::AtDREB1A-31, pRD29A::AtDREB1A transgenic line 31. BD, before drought; AD, after six days of drought.

Annotation using the COG (Cluster of Orthologous Groups of proteins) database showed that many DEGs in both comparisons were not annotated accurately and hence placed in the “general function prediction only” cluster. Considering the number of annotated genes in each category, “translation, ribosomal structure and biogenesis”, “secondary metabolites biosynthesis, transport and catabolism”, “posttranslational modification, protein turnover, chaperones” and “signal transduction mechanisms” were the top four categories for DEGs in the WT (BD) versus pRD29A::AtDREB1A-31 (BD) comparison using the COG database (Figure 5A), while “transcription”, ‘signal transduction mechanisms”, “carbohydrate transport and metabolism” and “replication, recombination and repair” were the top four categories in the WT (AD) versus pRD29A::AtDREB1A-31 (AD) comparison (Figure 5C). These results suggest DEGs in these categories are important in drought stress responses in transgenic *S. miltiorrhiza* plants expressing *AtDREB1A*.

**Figure 6:**
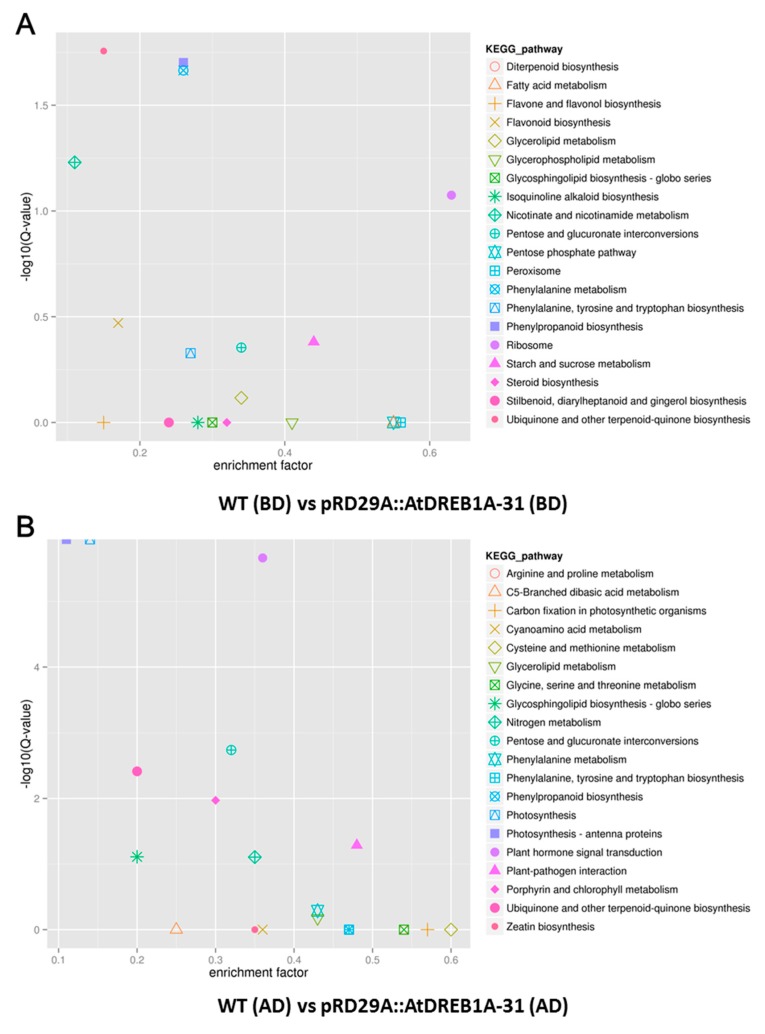
Scatter plot of KEGG pathways enriched in the comparisons of WT and *AtDREB1A* transgenic plants before and after drought treatment. (**A**) WT (BD) versus pRD29A::AtDREB1A-31 (BD); (**B**) WT (AD) versus pRD29A::AtDREB1A-31 (AD). WT, wild type; pRD29A::AtDREB1A-31, pRD29A::AtDREB1A transgenic line 31. BD, before drought; AD, after six days of drought.

**Figure 7:**
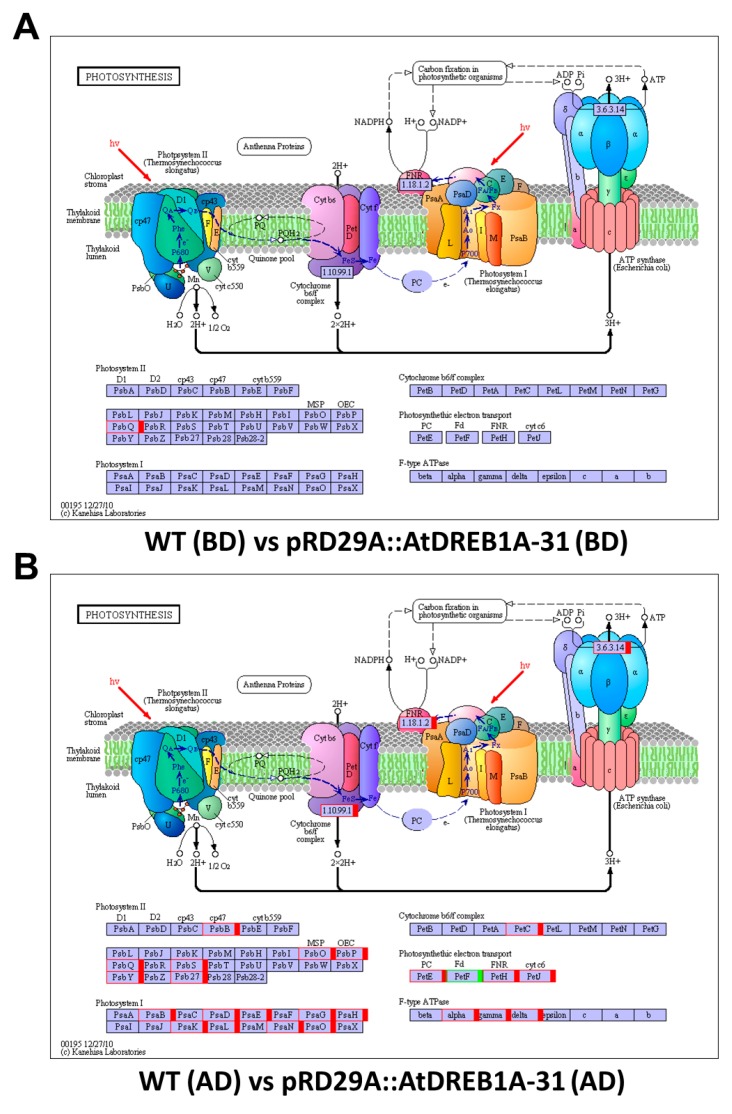
Analysis of DEGs related to photosynthesis in the comparisons of WT and *AtDREB1A* transgenic plants before and after drought treatment. (**A**) WT (BD) versus pRD29A::AtDREB1A-31 (BD); (**B**) WT (AD) versus pRD29A::AtDREB1A-31 (AD). Red/green indicate up- and down-regulated DEGs, respectively. Blue indicates DEGs with mixed patterns of regulation. WT, wild-type; pRD29A::AtDREB1A-31, pRD29A::AtDREB1A transgenic line 31. BD, before drought; AD, after six days of drought.

Kyoto Encyclopedia of Genes and Genomes (KEGG) identifiers were searched to predict biochemical pathways associated with DEGs. The top 50 enriched KEGG pathways associated with DEGs from the WT (BD) versus pRD29A::AtDREB1A-31 (BD) and WT (AD) versus pRD29A::AtDREB1A-31 (AD) comparisons are shown in Figure 5B,D, respectively. The pathway with the largest numbers of DEGs was “ribosome” in both comparisons (WT (BD) versus pRD29A::AtDREB1A-31 (BD) and WT (AD) versus pRD29A::AtDREB1A-31 (AD)). Furthermore, many DEGs from the WT (AD) versus pRD29A::AtDREB1A-31 (AD) comparison were enriched in the KEGG pathways “photosynthesis-antenna proteins”, “photosynthesis” and “plant hormone signal transduction” with relatively lower enrichment factors and higher Q-values (Figure 6). Before drought treatment, there was only one DEG in the photosynthesis pathway in the WT and pRD29A::AtDREB1A-31 comparison, while 30 DEGs were found in the WT (AD) versus pRD29A::AtDREB1A-31 (AD) comparison and most of these genes were upregulated in pRD29A::AtDREB1A-31 transgenic plants (Figure 7). 

**Figure 8:**
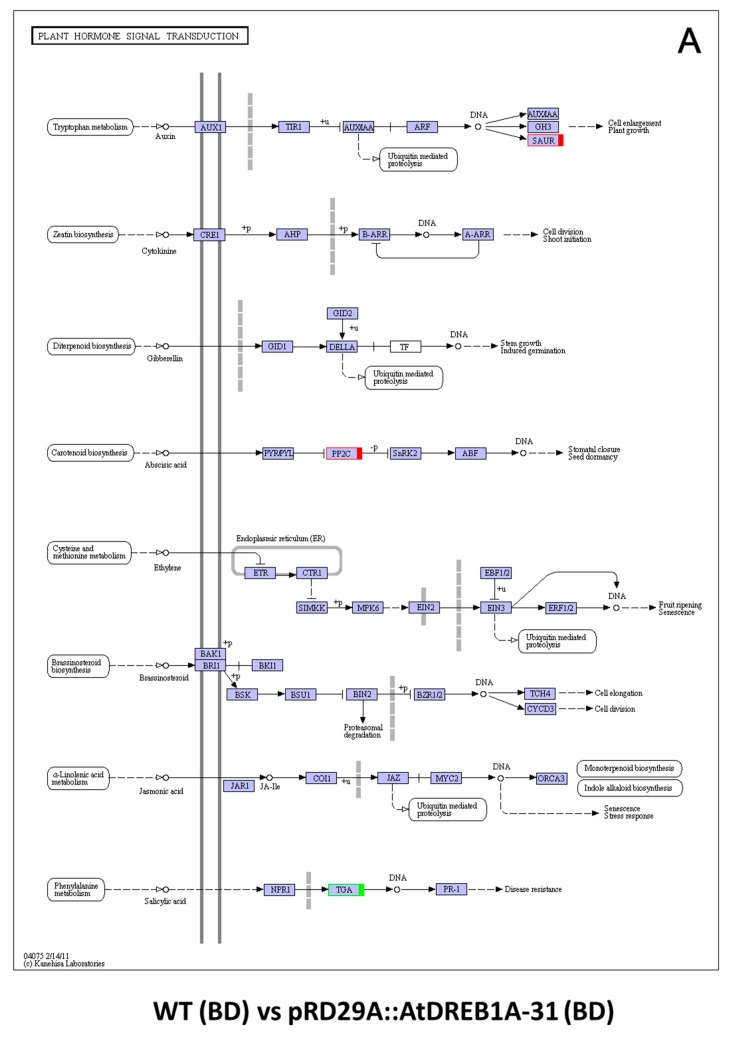

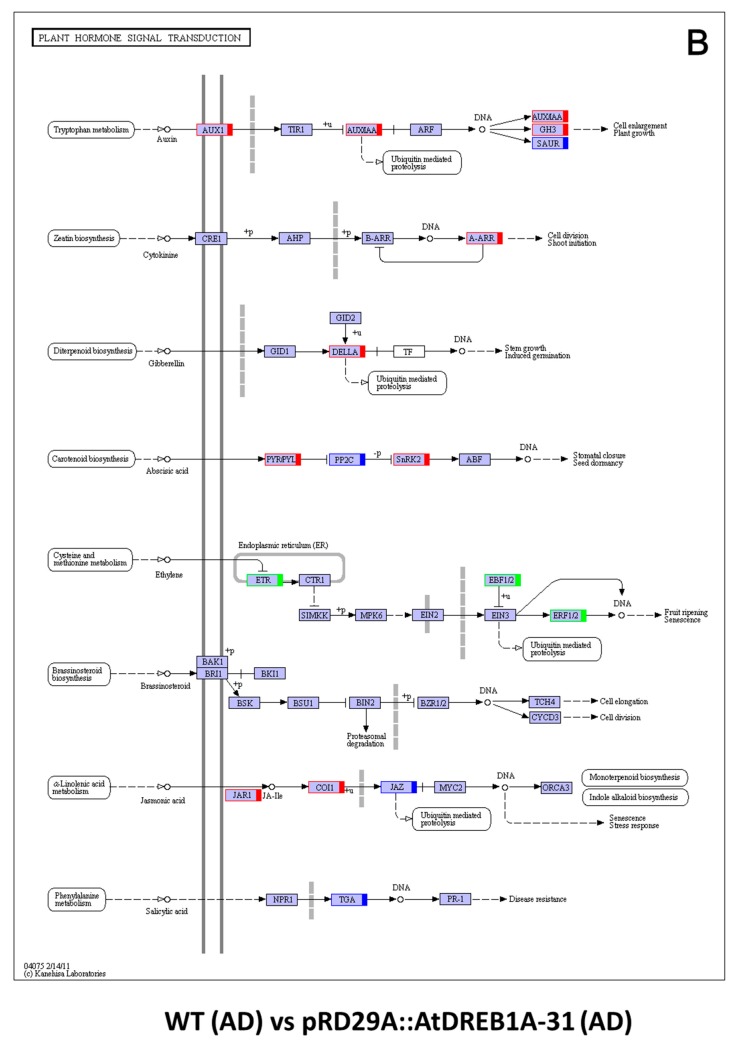
Analysis of DEGs related to plant hormone signal transduction in the comparisons of WT and *AtDREB1A* transgenic plants before and after drought treatment. (**A**) WT (BD) versus pRD29A::AtDREB1A-31 (BD); (**B**) WT (AD) versus pRD29A::AtDREB1A-31 (AD). Red/green indicate up- and down-regulated DEGs, respectively. Blue indicates DEGs with mixed patterns of regulation. WT, wild-type; pRD29A::AtDREB1A-31, pRD29A::AtDREB1A transgenic line 31. BD, before drought; AD, after six days of drought.

Plant hormone regulatory pathways including the salicylic acid, jasmonic acid, brassinosteroid, ethylene, ABA, gibberellin, cytokinin and auxin pathways, showed little change in the WT (BD) versus pRD29A::AtDREB1A-31 (BD) comparison, in which the *SAUR* and *PP2C* genes were up-regulated and *TGA* was down-regulated in the pRD29A::AtDREB1A-31 transgenic line (Figure 8A). Following exposure to drought stress, various pathways other than the brassinosteroid pathway are likely involved in AtDREB1A-mediated enhanced tolerance to drought in transgenic *S. miltiorrhiza* plants (Figure 8B). Analysis of plant hormone signal transduction pathways showed that two DEGs in the cytokinin pathway and four DEGs in the gibberellin pathway were all up-regulated and four DEGs in the ethylene pathway were all down-regulated in the pRD29A::AtDREB1A-31 transgenic plants, while the DEGs in the other pathways exhibited mixed patterns of expression (Figure 8B, Table S5).

### 2.4. DEGs Related to Transcription Factors

Gene expression was elevated or diminished upon introduction of the *AtDREB1A* transgene before and after drought treatment in five main stress-related transcription factor families, namely AP2/ERF (ethylene response factor), bZIP, MYB, NAC and WRKY. We identified 37 DEGs encoding AP2/ERF transcription factors, 11 DEGs encoding bZIP transcription factors, 23 DEGs encoding MYB-related proteins, 17 DEGs encoding NAC domain-containing proteins and 32 DEGs encoding WRKY transcription factors (Table S6, Sheet 1). Compared with the before-drought treatment, the number of DEGs between WT and the pRD29A::AtDREB1A-31 transgenic line associated with different transcription factor families increased after drought stress, especially in the AP2/ERF, bZIP and MYB families (Table S6).

### 2.5. Validation of DEGs by qRT-PCR Analysis

**Figure 9:**
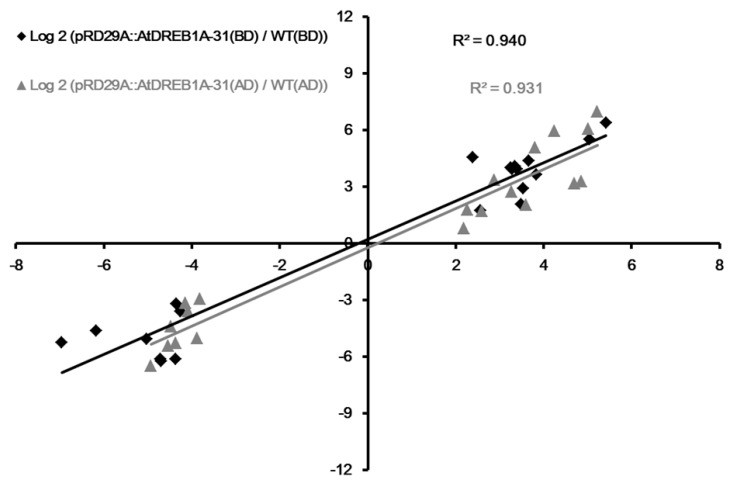
Correlations in changes in gene expression between fold-change determined from RNA-seq data (X-axis) and data obtained using qRT-PCR (Y-axis). WT, wild-type; pRD29A::AtDREB1A-31, pRD29A::AtDREB1A transgenic line 31. BD, before drought; AD, after six days of drought.

To validate the expression profiling results, we selected for further analysis 30 and 20 genes that were significantly up- and down-regulated in *AtDREB1A*-expressing transgenic plants, respectively, both before and after drought treatment (Tables S7 and S8). Quantitative RT-PCR was performed on 20 genes (12 and 8 significantly up- and down-regulated, respectively) selected at random among genes for which expression was altered in the transgenic line relative to WT plants before and after drought treatment (Table S9). The acquired relative expression levels of the selected genes were comparable between the two experimental methods (Figure S1) and a high correlation (R^2^ > 0.93) was observed between qRT-PCR and RNA-seq data (Figure 9), confirming the accuracy of the approach.

## 3. Discussion

In our previous study, we found that overexpression of *AtDREB1A* confers drought tolerance in transgenic *S. miltiorrhiza* plants. Because AtDREB1A is a transcription factor, we can predict that drought tolerance will result from changes to the transcriptional network of the plants [23]. To confirm this hypothesis and to gain new insight into the molecular mechanisms of the enhanced drought tolerance, global transcriptional profiling of WT and the *AtDREB1A*-expressing transgenic line was compared using RNA-sequencing (RNA-seq) technology in the present study. RNA-seq is a high-throughput sequencing technology that enables the study of global transcriptional profiling and has been widely used investigate molecular responses to abiotic and biotic stresses and for comparing transcriptomes under different treatments [4,11,28,29,30,31,32,33,34].

In the current study, we generated 22.96 Gb of clean sequencing read data that was assembled de novo into 78,915 unigenes (Table S2). Of these, 37,979 unigenes (~48% of assembled unigenes) were functionally annotated against public protein databases (COG, KOG, GO, KEGG, Swiss-Prot, Pfam and NCBI nr database). Thus, functional annotation could not be assigned for 52% of the assembled unigenes, due either to a match with a protein of unknown function, or because no matching homologous protein sequence was identified (Table S3). These unigenes could be of great interest, since they may be novel transcripts or alternatively spliced variants. 

Analysis of RNA-seq data revealed that the global gene expression profile of *S. miltiorrhiza* plants overexpressing *AtDREB1A* differed from that of control plants. Compared with WT, before drought, 1359 genes showed significantly different levels of expression in the pRD29A::AtDREB1A transgenic line, of which 423 were up-regulated and 936 were down-regulated. After six days of drought treatment, the number of DEGs increased to 2893, with 1580 up-regulated and 1313 down-regulated in the pRD29A::AtDREB1A transgenic plants, which indicated that AtDREB1A may mediate drought tolerance by modulating the expression of many stress-related genes. Interestingly, only 348 DEGs were shared between the before- and after-drought comparisons (Figure 3). This could be due to variations in the expression levels of the AtDREB1A transcription factor before and after drought stress, resulting in different genetic regulatory patterns in transgenic plants.

The ribosome is a macromolecular assembly that is responsible for protein biosynthesis in all organisms [35]. Some ribosomal proteins are known to play other important roles in plants. The multifunctional ribosomal protein S3 is a structural and functional component of the ribosome and DNA repair enzyme in the DNA base excision repair pathway [36]. The wheat ribosomal protein L5 gene has been shown to be dramatically induced by salt, freezing and drought stresses, suggesting that this gene may contribute to salt tolerance [37]. In this study, the “ribosome” pathway ranked first among the top 50 KEGG pathways of DEGs identified in the WT versus pRD29A::AtDREB1A-31 comparison both before and after drought stress (Figure 5), indicating that the AtDREB1A transcription factor can respond to drought stress by regulating the ribosome pathway in transgenic *S. miltiorrhiza* plants.

Transcription factors and phytohormones play an important role in the plant stress response [38,39,40,41]. Transcription factors are known to interact with cis-transcriptional regulatory elements such as promoters, silencers, enhancers, insulators and LCR regions that are situated adjacent to the genes that they regulate and they consequently regulate the expression of numerous downstream genes to control diverse biological processes [1,42,43]. A slight alteration in the relative abundance of transcription factor mRNAs can trigger reaction cascades that influence many physiological processes, resulting in major changes in downstream gene expression [44]. Several families of transcription factors including the AP2/ERF, bZIP, MYB, NAC and WRKY families are critical components of the plant adaptive response to abiotic stress [39,45,46]. In the current study, genes encoding members of the AP2/ERF, NAC and WRKY families were either significantly induced or suppressed in transgenic *S. miltiorrhiza* plants expressing *AtDREB1A* before drought stress; moreover, the number of DEGs classified in all five transcription factor families increased after drought stress (Table S6, Sheet 1).

Plant hormones are critical for allowing plants to adapt to environmental changes by mediating growth, development, nutrient allocation and source/sink transitions [40,47,48,49,50]. Although ABA is the most actively studied stress-responsive hormone, other phytohormones such as CK, ethylene, SA and JA in the response to environmental stresses are beginning to be better understood [49,51,52]. Recent evidence suggests plant hormones are involved in multiple processes and crosstalk between disparate plant hormone signaling pathways leads to synergetic and/or antagonistic interactions crucial for abiotic stress responses [53,54,55]. Based on our RNA-seq data, various pathways other than the brassinosteroid pathway are presumably involved in AtDREB1A-mediated enhanced drought tolerance in transgenic *S. miltiorrhiza* plants (Figure 8B). Additionally, many studies have shown that transcription factors and plant hormone signal transduction pathways interact cooperatively in the response to biotic and abiotic stresses [45,56]. Plant hormones such as SA, JA and ethylene regulate the expression of genes in the NAC and ERF transcription factor families that regulate various disease resistance pathways [1,57,58]. DREB proteins and DRE/CRT elements reportedly function in the ABA-dependent pathway [56] and specific ERF proteins, such as TSRF1, act as molecular nodes for the integration ABA and ethylene signaling pathways [59].

Photosynthesis in chloroplasts is particularly sensitive to stress and is a major source of cellular reactive oxygen species (ROS) [60]. ROS can irreversibly damage photosynthetic components, for instance by inhibiting restoration of the PSII complex by suppressing synthesis of the D1 protein. Thus, the increased capacity of transgenic lines to recover from drought-enhanced photoinhibition may be due to reduced accumulation of ROS [61]. Our previous studies showed that *AtDREB1A*-expressing transgenic *S. miltiorrhiza* plants displayed higher SOD, CAT and POD activities than WT plants, coupled with lower MDA levels and achieved a higher photosynthetic rate following drought stress, which confirmed this hypothesis [23]. In the present study, RNA-seq data revealed that expression of 29 photosynthesis-related genes was significantly up-regulated in transgenic versus WT plants following drought stress (Figure 7), suggesting AtDREB1A improved the photosynthetic capacity by regulating the expression of genes involved in this function. Furthermore, many genes that encode specific types of proteins, such as the late embryogenesis abundant protein (LEA), aquaporin and proline-rich proteins, were significantly up-regulated in transgenic plants [23], suggesting AtDREB1A may effectively regulate multiple genes in stress response pathways.

## 4. Materials and Methods

### 4.1. Plant Materials and Growth Conditions

WT *Salvia miltiorrhiza* plants (Zhongjiang, Sichuan) and the transgenic *S. miltiorrhiza* line pRD29A::AtDREB1A-31 (line 31) expressing the *AtDREB1A* gene under the control of the stress-induced RD29A promoter—which was obtained from our previous study [23]—were used in this experiment. To probe drought tolerance, WT and transgenic *S. miltiorrhiza* plants in sterile tubes on solid MS medium were removed and placed in sterile soil with appropriate nutrients and cultured at 25 ± 2 °C in a growth chamber with a photoperiod of 16 h:8 h (light intensity = 50 μmol m^−2^ s^−1^). Prior to drought stress, pots of drought-stressed and well-watered plants were saturated with water and placed overnight to drain. Plants at uniform stages of development were then selected for stress treatments [23]. On day 25 (before drought) and day 31 (after six days of drought), three individual plants of transgenic *S. miltiorrhiza* line pRD29A::AtDREB1A-31 and wild type were collected for RNA extraction.

### 4.2. RNA Isolation, Library Construction and RNA Sequencing

Total RNA was extracted from mixed samples consisting of three separate plants by using a modified CTAB method [23]. Residual genomic DNA was eliminated using RNase-Free DNase I (New England BioLabs, Beverly, MA, USA). After characterization of RNA purity using a Nanodrop1000 spectrophotometer (Thermo Fisher Scientific, Wilmington, DE, USA) and following the measurement of RNA concentration using a Qubit 2.0 Fluorimeter (Life Technologies, Carlsbad, CA, USA), the integrity of RNA was investigated using an Agilent Bioanalyzer 2100 system (Agilent Technologies, Santa Clara, CA, USA). The library was constructed using RNA samples with an integrity number > 7.0 and Biomarker Technologies Corporation (Beijing, China) performed Illumina RNA sequencing following procedures similar to those described previously by Zhang et al. [62]. After enrichment and purification with oligo (dT) paramagnetic beads, mRNAs were sheared into short fragments that were subsequently used as templates for first- and second-strand cDNA synthesis. AMPure XP beads were employed for purifying double-stranded cDNA, 3′ ends were enzymatically repaired, polyadenylated and ligated to adapters to select templates of different sizes and the four resulting cDNA libraries were subjected to PCR amplification and sequenced using an Illumina HiSeq2500 RNA sequencing instrument.

### 4.3. De novo Transcriptome Assembly and Functional Annotation

After removing low quality RNA-seq reads, reads consisting only of adaptors and reads with >5% unknown nucleotides (Ns), clean reads were filtered from raw reads [63]. De novo assembly was carried out using a Trinity assembler, and group pair and K-mer distances were set at 300 and 25, respectively, and default values were used for all other parameters. Longer contigs were assembled from short reads based on overlapping regions and clustered and further assembled into unigenes by considering paired-end information [62]. 

The predicted unigene protein sequences were aligned to selected protein databases using BLASTx (*E*-value ≤ 10^−5^); these included the Kyoto Encyclopedia of Genes and Genomes (KEGG, http://www.genome.ad.jp/kegg/), the National Center for Biotechnology Information (NCBI) non-redundant (nr) protein database (ftp://ftp.ncbi.nih.gov/blast/db) and Clusters of Orthologous Groups (COGs, http://www.ncbi.nlm.nih.gov/COG). Gene Ontology (GO, http://www.geneontology.org) terms describing molecular functions, biological processes and cellular components were assigned to predicted genes using Blast2GO based on the outputs from Nr BLASTp searches [2]. Functional classification of COG and GO terms was performed for all genes using in-house Perl scripts [1].

### 4.4. Identification of DEGs

Clean reads were mapped to the unigene library with Bowtie and the read count of each gene was derived by mapping the results using RSEM. Fragments Per Kilobase of transcript per Million mapped reads (FPKM) values for each unigene were calculated to determine expression profiles and differences in gene expression between WT and transgenic plants were analyzed using DESeq, by employing the Benjamini and Hochberg False Discovery Rate (FDR) method. DEGs were identified using FDR < 0.01 and Fold-Change (FC) ≥ 2 as thresholds and cluster analysis was carried out based on differential expression of unigenes across samples [62].

### 4.5. Validation of DEGs by qRT-PCR

In order to confirm the accuracy of expression profiles derived from the RNA-seq experiments, 40 genes were selected for confirmation by quantitative RT-PCR (qRT-PCR) assays. RNA (2 μg) from each sample was treated with RNase-Free DNase I (New England BioLabs) and a High Capacity cDNA Reverse Transcription Kit (Takara) was used for cDNA synthesis following the manufacturer’s instructions. All cDNA samples were quantified on a Nanodrop1000 spectrophotometer [64].

The qRT-PCR assays were performed on an iQ5.0 instrument (Bio-Rad, Hercules, CA, USA) in combination with a SYBR Green qPCR kit (Roche, Basel, Switzerland) by heating at 95 °C for 3 min, followed by 40 cycles at 95 °C for 30 s and 60 °C for 30 s. Dissociation curves at the end of each run were used for monitoring amplicon specificity with amplification reactions (20 μL) consisting of 10 μL 2×SYBR Green Mix, 1 μL cDNA and 0.25 μM forward and reverse primers. Relative gene expression levels were determined by employing the 2^−ΔΔ*C*t^ method and normalized against *GAPDH* and *Actin*. All assays were performed in triplicate under identical conditions and correlations between qRT-PCR and RNA-seq data were evaluated using Pearson correlation coefficients [23].

## 5. Conclusions

**Figure 10:**
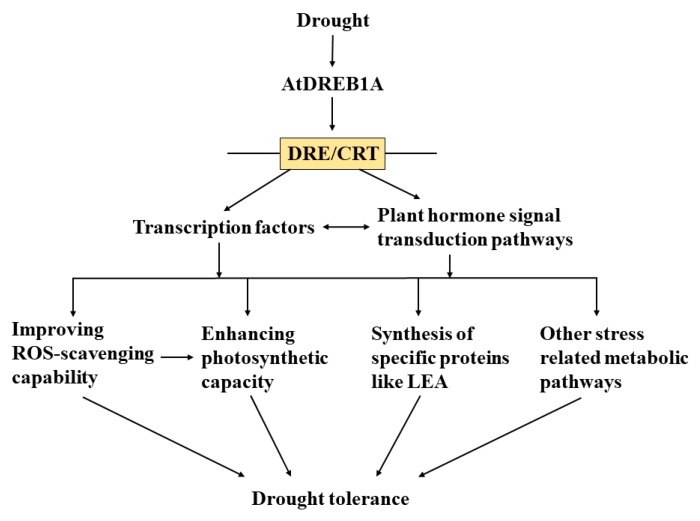
Proposed model for AtDREB1A function during drought stress.

Based on our previous and current results, we can propose a simple model to explain the increased drought tolerance of *AtDREB1A*-overexpressing plants of *S. miltiorrhiza* (Figure 10): (1) drought stress induces the expression of AtDREB1A; (2) The activated AtDREB1A protein then binds to DRE/CRT sequence motifs to regulate the expression of its target genes; (3) These genes directly or indirectly activate transcription factors and plant hormone signal transduction pathways, which result in the activation of different downstream pathways; (4) Thereafter, genes with significantly altered expression,—such as those encoding SODs, PODs, PsbX and LEA—trigger physiological changes including reduced ROS accumulation, enhanced photosynthetic capacity and elevated LEA levels, which ultimately result in improved drought stress tolerance in transgenic plants.

## Supplementary Materials

Supplementary materials can be found at http://www.mdpi.com/1422-0067/19/3/827/s1.

## Abbreviations

ABA	abscisic acid
CAT	catalase
CK	cytokinin
CTAB	cetyltrimethyl ammonium bromide
DRE	dehydration response element
FC	fold change
JA	jasmonic acid
LEA	late embryogenesis abundant protein
MDA	malondialdehyde
POD	peroxidase
ROS	reactive oxygen species
RWC	relative water content
SA	salicylic acid
SOD	superoxide dismutase
