# Supplementary material for: Comparative Transcriptome Analyses Reveal Potential Mechanisms of Enhanced Drought Tolerance in Transgenic Salvia Miltiorrhiza Plants Expressing AtDREB1A from Arabidopsis
Source: Int J Mol Sci. 2018 Mar 12;19(3):827. doi: 10.3390/ijms19030827 (PMC5877688; doi:10.3390/ijms19030827)
Supplement: Supplementary file 1 [file ijms-19-00827-s001.pdf]

Table S1 Sequencing data statistics for the four *S. miltiorrhiza* RNA-seq libraries.

Samples	Read Number	Base Number	GC Content	%≥Q30
WT (BD)	27,026,927	6,809,764,858	49.35%	88.14%
pRD29A::AtDREB1A-31 (BD)	23,323,827	5,876,581,426	49.90%	87.87%
WT (AD)	23,222,857	5,851,276,192	48.74%	87.67%
pRD29A::AtDREB1A-31 (AD)	17,566,704	4,425,994,270	49.75%	87.19%

Table S2 Assembly results for the *Salvia miltiorrhiza* transcriptome.

Length Range	Contig	Transcript	Unigene
200-300 nt	11,973,364(99.53%)	37,100(21.43%)	31,158(39.48%)
300-500 nt	23,458(0.19%)	27,410(15.83%)	18,814(23.84%)
500-1000 nt	16,990(0.14%)	30,708(17.74%)	13,567(17.19%)
1000-2000 nt	10,769(0.09%)	41,782(24.13%)	9,124(11.56%)
≥2000 nt	5,787(0.05%)	36,129(20.87%)	6,252(7.92%)
N50 Length	42	2,048	1,301
Mean Length	41.52	1230.65	711.83
Total Number	12,030,368	173,129	78,915
Total Length	499,557,119	213,061,724	56,173,939

Table S3 Summary of Unigene annotations from seven public databases.

Databases	Annotated sequence	300≤length<1000	length≥1000
COG	13,904	4,417	6,092
GO	27,694	9,738	11,250
KEGG	10,017	3,658	3,419
KOG	22,861	7,958	8,682
Pfam	26,117	8,871	12,254
Swiss-Prot	26,052	9,587	10,753
NR	37,091	13,680	14,106
Total	37,979	13,974	14,139

Table S4 Statistics of differentially expressed genes (DEGs) in the two comparisons before and after drought.

DEG Set	All DEGs	Up regulated	Down regulated
WT (BD) vs pRD29A::AtDREB1A-31 (BD)	1,359	423	936
WT (AD) vs pRD29A::AtDREB1A-31 (AD)	2,893	1,580	1,313

Table S5 Classification of differentially expressed genes (DEGs) related to plant hormone signal transduction in WT and *AtDREB1A*-expressing transgenic *Salvia miltiorrhiza* plants after drought stress.

Plant hormone	Unigenes identified	Total DEGs	Up regulated	Down regulated
Auxin signaling	81	11	10	1
Cytokinine signaling	21	2	2	0
Gibberellin signaling	12	4	4	0
Abscisic acid signaling	44	5	3	2
Ethylene signaling	20	4	0	4
Jasmonic acid signaling	18	7	3	4
Salicylic acid signaling	13	2	1	1

Table S6 Summary of differentially expressed genes (DEGs) related to transcription factor families in *AtDREB1A*-expressin transgenic *Salvia miltiorrhiza* compared to WT.

Transcription factors	Unigenes identified	DEGs	Before drought			After drought		
			Up regulated	Down regulated	Total	Up regulated	Down regulated	Total
AP2/ERF	136	37	11	4	15	10	16	26
bZIP	56	11	1	0	1	4	7	11
MYB	144	23	0	1	1	10	12	22
NAC	93	17	1	7	8	0	12	12
WRKY	85	32	2	12	14	7	15	22

Table S7 Significant DEGs in the pRD29A::AtDREB1A-31 transgenic line compared to WT plants before drought treatment.

S.miltiorrhiza unigene ID	RefSeq ID	Description	WT (BD)	pRD29A::AtDREB1A-31 (BD)	Log2FC
**Significantly up regulated genes**					
c50672	CAA35093	Phosphinothricin acetyl transferase (Bar)	0.69	1297.65	10.72
c51153	At4g25480	AtDREB1A	0.07	29.85	7.99
c36680	XP_011096577.1	Protein HEADING DATE 3A	0.31	30.89	6.26
c51022	XP_009794994.1	PREDICTED: purple acid phosphatase 2-like	2.60	118.57	5.42
c36736	AJ130888	ABA-inducible protein	2.54	96.07	5.09
c34566	XP_012828897.1	PREDICTED: uncharacterized protein LOC105950126	1.20	44.72	5.04
c42747	AED92230	Stress-induced protein KIN1	0.82	23.25	4.61
c16127	ABP01769.1	Non-specific lipid-transfer protein 2	3.18	53.10	3.83
c29546	AKG51698.1	Polyphenol oxidase	1.28	19.62	3.67
c37325	ABU87404.1	SMLII	1.38	20.93	3.49
c47005	XP_006340524.1	Ethylene-responsive transcription factor ERF025	3.91	48.41	3.53
c46642	Q39529.1	Agglutinin-2	714.80	7930.29	3.39
c34211	XP_019196282.1	Acid phosphatase 1	74.09	792.40	3.34
c51626	XP_011088570.1	Dirigent-like protein	2.09	22.75	3.28
c43963	XP_009776612.1	PREDICTED: acid phosphatase 1-like	21.64	219.13	3.26
c41077	XP_011088570.1	Dirigent-like protein	4.01	26.15	2.61
c18143	XP_011083184.1	Dehydrin DHN1	38.30	237.97	2.56
c36895	ANF06979.1	Gibberellin-regulated protein 14	21.18	128.82	2.52
c29373	XP_012846750.1	Xyloglucan endo-transglycosylase	3.42	20.96	2.47
c18580	XP_012490174.1	PREDICTED: uncharacterized protein LOC105802843	5.58	32.36	2.44
c45424	XP_012073379.1	Probable plastidic glucose transporter 3	4.93	27.39	2.39
c34535	XP_012849481.1	Ethylene-responsive transcription factor ERF039	7.57	41.34	2.37
c49104	XP_012849532.1	PREDICTED: protein cfxQ homolog isoform X2	5562.34	29878.95	2.34
c50773	XP_002283016.3	Uncharacterized protein LOC100264228	9.75	48.21	2.22
c30437	XP_011099870.1	PREDICTED: spartin-like isoform X2	7.98	33.72	1.99
c44715	NP_198686.2	Cotton fibre expressed protein	23.29	96.91	1.98
c45423	XP_011082560.1	Patatin-like phospholipase	6.60	26.01	1.90
c15852	XP_012837236.1	Expansin-like A2	31.87	124.04	1.88
c27954	XM_015163624.1	Drosophila mojavensis uncharacterized protein	51.54	198.92	1.87
c17334	XP_012836538.1	PREDICTED: uncharacterized protein LOC105957157	6.53	25.08	1.85
**Significantly down regulated genes**					
c50771	WP_057766412.1	RNA polymerase sigma factor SigB	72-44	7.43	-3.30
c50915	XP_019264644.1	Putative lipid-transfer protein DIR1	37.50	3.50	-3.48
c18544	AAY68291.1	Lectin (Precursor)	32.19	2.79	-3.56
c40726	XP_011071353.1	Probable protein Pop3	97.14	7.66	-3.72
c44918	XP_011097626.1	Nitrate reductase	45.52	3.31	-3.85
c44813	XP_011085796.1	Metalloendoproteinase 1	90.84	6.05	-3.96
c45727	XP_011099650.1	Germin-like protein subfamily 1 member 13	156.84	9.64	-4.10
c40649	AFN85535.1	PRp27-like protein	90.49	5.21	-4.18
c46479	XP_011071279.1	Subtilisin-like protease	21.98	1.18	-4.27
c36817	AEM97876.1	Chitinase 5	385.13	19.71	-4.36
c43911	XP_011075887.1	PREDICTED: hyoscyamine 6-dioxygenase	38.03	1.90	-4.37
c35004	XP_012840657.1	Metalloendoproteinase 1	52.08	1.93	-4.71
c45895	KMS98576.1	hypothetical protein BVRB_4g092630	25.81	0.94	-4.72
c51454	AFK26309.1	Chitinase 3	41.19	1.47	-4.84
c18197	XP_012836300.1	Thaumatin-like protein (Precursor)	856.39	27.52	-5.03
c19499	AAM21199.1	Pathogenesis-related protein 5-1	102.63	1.99	-5.71
c16149	XP_011083774.1	Glucan endo-1,3-beta-glucosidase	560.44	8.14	-6.18
c39931	XM_011071550.1	Sesamum indicum EG45-like domain containing protein	656.45	5.47	-6.96
c50692	XP_011092473.1	Pathogenesis-related leaf protein 6	784.09	2.05	-8.61
c48549	YP_002647021.1	RNA-dependent RNA polymerase	12201.08	7.62	-10.72

Table S8 Significant DEGs in the pRD29A::AtDREB1A-31 transgenic line compared to WT plants after drought stress.

S.miltiorrhiza unigene ID	RefSeq ID	Description	WT (AD)	pRD29A::AtDREB1A-31 (AD)	Log2FC
**Significantly up regulated genes**					
c51153	At4g25480	AtDREB1A	0	85.70	10.15
c50672	CAA35093	Phosphinothricin acetyl transferase (Bar)	1.60	1707.66	9.48
c36736	AJ130888	ABA-inducible protein	0.72	72.03	5.82
c33938	LOC101254950	Proline-rich protein 4	8.41	460.03	5.22
c18558	NP_181593	Rare lipoprotein A (RlpA)-like	0.40	28.39	5.20
c40729	LOC100250504	Chloroplast chlorophyll a/b binding protein	115.91	5448.49	5.02
c27605	AT5G10570	Transcription factor bHLH61 isoform 1	0.16	11.63	4.92
c44834	Q9SN46	Leucine-rich repeat extensin-like protein 5	0.71	31.69	4.88
c16091	At5g54270	Chlorophyll a-b binding protein of LHCII type III	6.83	305.61	4.85
c36589	AT4G38770	Proline-rich protein 4	14.89	562.51	4.69
c47079	XP_002284828	Auxin responsive protein	0.66	30.28	4.66
c16109	Q39529	Agglutinin-2	30.52	1088.61	4.62
c35965	T28I19.80	Xyloglucan endotransglycosylase	2.69	90.30	4.50
c18785	XM_007034296	Gibberellin-regulated family	0.31	12.23	4.24
c28876	AT2G06520	Photosystem II reaction centre X protein (PsbX)	7.75	196.28	4.11
c50673	AT5G54270	LHC II Type III chlorophyll a /b binding protein	9.58	236.02	4.02
c18256	EOY07369	Hydrophobic seed protein	3.37	80.88	3.98
c36119	AT2G47450	Signal recognition particle 43 kDa protein	1.80	37.52	3.81
c18229	LOC100259814	Glyceraldehyde-3-phosphate dehydrogenase B	4.80	95.02	3.73
c15119	LOC101244541	Stress-induced protein KIN2	3.69	73.47	3.72
c36069	AT1G25440	Zinc finger protein CONSTANS-LIKE 16	6.42	113.93	3.61
c42747	AED92230	Stress-induced protein KIN1	3.73	59.12	3.42
c29058	AED94334	Proline-rich receptor-like protein kinase	30.37	449.89	3.35
c42325	EOY33466	Dienelactone hydrolase family	5.31	75.22	3.27
c37798	AT4G36530	Alpha/beta hydrolase family	2.06	28.03	3.19
c33378	LOC100243277	Geranylgeranyl diphosphate reductase	31.27	322.75	2.88
c30457	AT1G68520	B-box zinc finger	3.41	30.93	2.59
c42086	AT5G13160	Serine/threonine-protein kinase PBS1	2.34	16.57	2.34
c40320	At3g01090	SNF1-related protein kinase catalytic subunit alpha KIN10	1.30	9.29	2.27
c50102	AT1G51140	Transcription factor bHLH122	9.05	59.67	2.18
**Significantly down regulated genes**					
c52057	XP_002272245.2	Uncharacterized protein LOC100247337	21.16	2.58	-3.36
c50693	XP_011085010.1	Delta-1-pyrroline-5-carboxylate synthase	975.42	139.20	-3.35
c16463	XP_012853757.1	Cytochrome P450 81D1	48.54	6.39	-3.40
c38529	ADP06653.1	Malate synthase	78.41	10.69	-3.41
c45529	XP_012856043.1	Premnaspirodiene oxygenase	77.31	10.27	-3.44
c50097	ABC60435.1	Cytoplasmic actin	36.14	4.51	-3.47
c36883	ADB43614.1	Alcohol dehydrogenase 2	220.78	27.62	-3.53
c46119	EEF40726.1	Auxin:hydrogen symporter, putative	62.94	7.69	-3.56
c40774	XP_011092960.1	PREDICTED: zeaxanthin epoxidase, chloroplastic-like	57.77	5.89	-3.82
c43520	XR_001172948.1	Uncharacterized protein LOC105959466	22.86	2.17	-3.88
c44334	XP_012850744.1	PREDICTED: cytochrome P450 CYP72A219-like	19.80	1.63	-4.09
c15647	XP_011097089.1	NAC transcription factor	44.21	3.56	-4.14
c30081	XP_011100326.1	Myb-related protein Myb4	19.43	1.37	-4.24
c45305	AGW27192.1	Tyrosine aminotransferase 3	16.09	1.04	-4.36
c15279	EOY06612.1	MLP-like protein 423	68.88	4.37	-4.47
c48388	XP_012834490.1	MLO-like protein 6	341.74	21.13	-4.55
c15293	XP_011095083.1	Aldose reductase	73.10	3.39	-4.93
c50848	CAP72045.1	Tubulin alpha-2	21.39	0.12	-6.81
c51647	AAA96144.1	Destabilase	39.41	0.29	-6.85
c48549	YP_002647021.1	RNA-dependent RNA polymerase	30212.32	20.09	-11.09

Table S9 Oligonucleotide primers used in this study.

Oligo name	Sequence (5' to 3')	Oligo name	Sequence (5' to 3')	Oligo name	Sequence (5' to 3')
c51022-F	CACACGGTGCGGACTTTCTG	c34566-F	CAGCCTCCAATCCACCAACA	c16127-F	GGCGAGGGATTTCAGGCAGTT
c51022-R	GCTCGGGTTCGACTCGTAATG	c34566-R	TCCGACAACACCGCTCACA	c16127-R	GCGAGTGAGGCCGCGATAA
c29546-F	ACATTGACATCGGCGACCAG	c37325-F	AGGAAGAAAGCCGCTCAAATC	c47005-F	AGTGCATGGCTGCTGAAGACG
c29546-R	TCACTACACGCATTGCTTCCC	c37325-R	GTCTAGGTCATCCCGAAACACG	c47005-R	ACCTCCGACACCCATTTACCG
c46642-F	GGATTTCGGTGTTCAAAGGC	c34211-F	CGGCAGCGACGACTACAAAG	c51626-F	TGAGTTGATGTCCGGCTCCTC
c46642-R	CCCCAAACCCTAATTCCTCTT	c34211-R	GCCTAGCAAGGTAGCCGAGGT	c51626-R	CGCAATCTCATGCCTTCTCGTA
c43963-F	CCCACCGACATAAGGACAAGC	c18143-F	GCCCACCATTTGCTACATACATT	c45424-F	TCCTATTTGGCTATCATCTCGGG
c43963-R	CCGATTCCCAACATTAGCACC	c18143-R	AGTCGGTTCCATGAGTAACCTTTC	c45424-R	CCAACGTGTCATTAACAACCCTG
c46479-F	CCGCCACGCTGTCTAAGTCA	c36817-F	CCTCGCCCTACTTCTCGTCG	c43911-F	CCTAAGGACCCATCAGAAGCC
c46479-R	AGCGCACGTAGCCATGTATAAAG	c36817-R	CCACTTTGACTCCATTGGTGGT	c43911-R	CAAAGTTCCAAGCAATGACCC
c35004-F	AGCCCTCGAATCAGCCATCA	c45895-F	CAGTTGGGCGAAGTCTATGGC	c18179-F	TTGTTGGGCTGGTTGAGGG
c35004-R	TGTCCGCCACTCCACAACG	c45895-R	GGGGGCAAGATGTAGCTCAGTA	c18179-R	CCGTCGTGGCAGGTAATCG
c16149-F	CGCCCAATAGACAACCCAAAT	c39931-F	GGCGGGTTATAGTCGATGAGG	c33938-F	GGCGATAGGTTGGGCTTGG
c16149-R	TCGTATCGCCTGAAGGTGAAAG	c39931-R	GCTACGGGTATGAAGACAAAGGC	c33938-R	CTCATCCACATTGTGCTCCTCC
c40729-F	TTAGACCTTCGCCAAGAACCG	c16091-F	GAGTGGAAGTCCCAGAAAGCG	c36589-F	GGCGGGAAGTGCCCGAATT
c40729-R	CCCAAATAATCAAGTCCACCCTC	c16091-R	GTTCTTCCTGGGGAGGTTCAAG	c36589-R	CCACCACCGATCACGAAGAAGC
c18785-F	TGTTGGGTGGTTGAGGCTGAG	c36119-F	TTCGCCGTAGGACTCTTCTTCA	c36069-F	AGCGTGGTTGGATGAATGGG
c18785-R	CGGCGTCCGATCCTTTGTT	c36119-R	TTCCACCCACTTCCGCTCA	c36069-R	GCTGCGTTGAGTTTCCTGACCT
c42325-F	GCAGAATCCAATGATCCCTAGC	c33378-F	CTTCTTGATGTCGCCCTTGTG	c30457-F	GCGGTGACGATGAGGTAGTCTTG
c42325-R	ACAAGCAGAAGCCAGAACACG	c33378-R	ACTTCTACGGCTGGGTGTTCC	c30457-R	CTTGCGACTCCTCCGTCCACT
c40320-F	GAGGAGGCACGCATGTTCTTTC	c50102-F	CGGAGGCGGTAACAGTGCTAAT	c40774-F	ACTTCATCTCATCTTCCGCTGC
c40320-R	GCCACGCATAATGTTACTCAAACC	c50102-R	CCCTCCAATGCCTTCTGAAATAG	c40774-R	ACCAGAGCCCTCCAACCATT
c43520-F	AAGATGGGCAGCAGGAGCAG	c44334-F	GGGTAAGGGTTCCCGTTGAG	c15647-F	GGTGAATGAAGGGTGGTGATG
c43520-R	GGGGGAAGAGGATGTTTCGTT	c44334-R	CCGTAGCTGCATCCATAGCAT	c15647-R	GTTCGGGTACTTGCGGTCTC
c45305-F	CAATCACGATAATGCCAAGCC	c15279-F	TAGGGTCTTGTATCATGGGTGTCA	c48388-F	TCGTCAGCATACGGGATACACC
c45305-R	CGAATCTCGCAGATCACAACAC	c15279-R	GGGGTTCATCTATGCGATTCTTC	c48388-R	CCCAAACTTTCCACTGCCTCA
c15293-F	GCCCATTTCACAAGCACCTG	Actin-F	GGTGCCCTGAGGTCCTGTT	GAPDH-F	CCACCGTCCACTCCATCACT
c15293-R	TTATCCACGTCACGGCCTACTC	Actin-R	AGGAACCACCGATCCAGACA	GAPDH-R	TGGGAACTCGGAACGACATAC

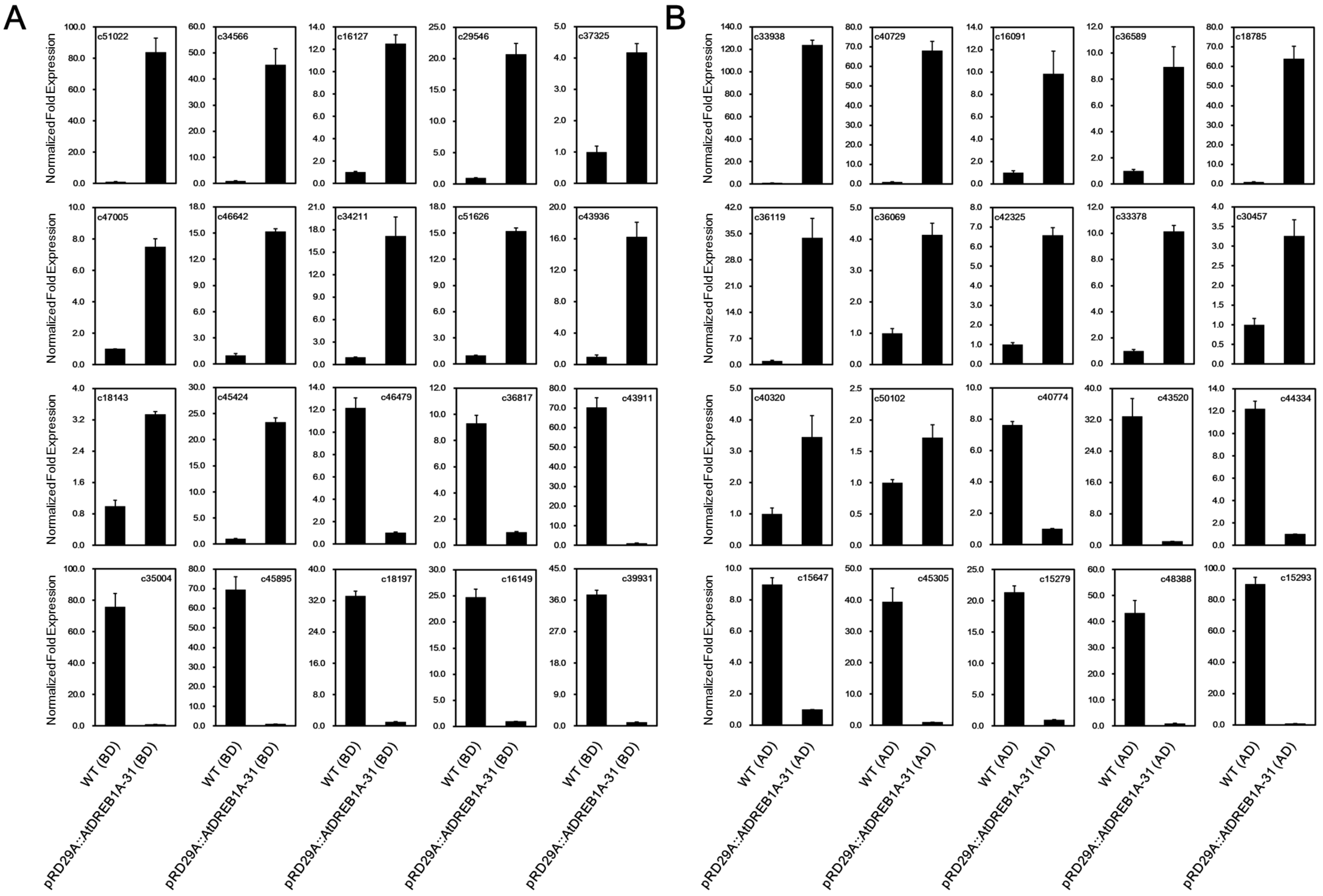

Figure S1 Validation of RNA-seq gene expression results by qRT-PCR analysis.
